# New York Notes

**Published:** 1900-01

**Authors:** 


### NEW YORK NOTES.

Something approaching a medical scandal was produced at the Columbus meeting of the American Medical Association by the effort, fortunately futile, to introduce a paper on the subject of a cure for tuberculosis, for which Crotte, of Paris, stood sponser. He has now come to the surface again at St. Luke's Hospital in New York, where it is stated that he is conducting voluminous experiments with a corps of nine physicians, not otherwise identified, as a jury. He seems to combine in his method of treatment static electricity and formaldehyde, and the results as they proceed, together with bacteriological cultures, are being filed with the city department of health, which, however, disclaims all responsibility.

In his recently published work, "The Modern Treatment of Wounds," Dr. John E. Summers, Jr., of Omaha, Neb., has followed an excellent system of presentation. In each case he outlines and defines the theater of operation, and gives working rules for the operations indicated.

A remarkable case is reported showing the extraordinary powers of the new anesthetic Chloretone, which is now being so much talked about. A man who, somehow or another, came to take a very excessive dose, namely, three dozen 3-gr. tablets, fell into a slumber, from which he did not waken for three days, and then experienced no untoward symptoms. The usual dose given in cases of insomnia is from one to three tablets.

Dr. Frederick Fraley, Jr., Philadelphia, reporting on a series of cases of gonorrhea which he has treated with mercurol, an organic compound of mercury with nuclein, containing about 10 per cent. of mercury, says that out of twelve cases six were absolutely cured in less than four weeks, three were practically cured within three weeks, and three were distinctly improved in a still shorter period. Of the latter one failed to report at the clinic, and the other two had complications which led to permanganate of potassium being used as supplemental treatment.

The subject of smallpox has lately been revived on account of the fatal prevalence of the disease among our troops in the Philippines. Dr. David P. Austin, who has conducted a large series of experiments on himself, besides having had a wide experience as a vaccinating officer of the Board of Health, says that soldiers and others likely to be exposed to infection should be immunized by repeated vaccination, as he himself has been. The great prevalence of varioloid in the Philippines as compared with Cuba and Porto Rico, he declares to be due to the fact that the soldiers out there were recruited for the most part in the far western States, where the regulations in regard to vaccination are very laxly enforced. He advises doctors to be careful about the lymph they use, the best being glycerinized and hermetically sealed in capillary tubes.

